# Protein Considerations for Athletes With a Spinal Cord Injury

**DOI:** 10.3389/fnut.2021.652441

**Published:** 2021-04-13

**Authors:** Joelle Leonie Flueck, Jill A. Parnell

**Affiliations:** ^1^Swiss Paraplegic Centre, Institute for Sports Medicine, Lucerne, Switzerland; ^2^Department of Health and Physical Education, Mount Royal University, Calgary, AB, Canada

**Keywords:** sports nutrition, spinal cord injury, paralympic athletes, amino acids, wheelchair athletes, dietary protein, performance nutrition

## Abstract

Athlete participation in the Paralympic games is steadily increasing; prompting research focused on the unique needs of this population. While the Paralympic Games includes a diversity of athletes, athletes with a spinal cord injury (PARA-SCI) represent a subgroup that requires specialized recommendations. Nutritional guidelines designed to optimize performance, in the context of the neurological impairments, are required. This narrative review summarizes the current literature regarding the importance of dietary protein for optimal health and performance. Factors with the potential to affect protein needs in PARA-SCI including loss of active muscle mass, reduced energy expenditure, and secondary complications are examined in detail. Furthermore, we analyze protein intakes in PARA-SCI from the available research to provide context around current practices and trends. In conclusion, we make the case that protein recommendations for able-bodied athletes may not be directly transferable to PARA-SCI. Consequently, PARA-SCI need their own guidelines to maximize performance and ensure long-term health.

## Introduction

Participation in Paralympic games is steadily increasing, with the events in London in 2012 receiving mass-media coverage for the first time (1) and the 2018 Games in PyeongChang reporting 567 athletes from 49 delegations, the most ever (2). Moreover, the Paralympic Games may motivate children and young people with disabilities to participate in sport, increasing their quality of life and social participation. Qualitative research found that children with disabilities were inspired by Paralympic athletes, and that the Paralympic games provided them with more confidence should they wish to participant in sport (1). Furthermore, the worldwide incidence of spinal cord injuries (SCIs) is estimated to be up to 500,000 individuals each year (3). All considered, the number of athletes with a SCI (PARA-SCI) is predicted to increase and the level of competition will intensify. While wheelchair athletes include a diverse group (4), the focus of this article is specifically on PARA-SCI, defined as those with paraplegia or quadriplegia participating actively in wheelchair sports. The reason of the injury could be traumatic or non-traumatic with consequences in the motor, sensory, and autonomic systems. In parallel with the growth in wheelchair sports is a need for nutritional recommendations specific to PARA-SCI that will promote health and optimize performance. Presently, this population is understudied, and evidence-based recommendations are lacking; creating uncertainty among athletes, coaches, and other support personnel. Research conducted to date indicates that PARA-SCI are at risk for multiple nutritional deficiencies (5), which can have a negative impact on their performance. Furthermore, recommendations specific to PARA-SCI are required, as those developed for able-bodied athletes may not be appropriate. Dietary protein is essential for athletes, as it has a role in energy production and synthesis of metabolic and contractile proteins (6, 7). Given the lack of recommendations for PARA-SCI, and the critical role of protein in exercise performance, the current article provides an overview of the physiology of PARA-SCI, considerations for protein recommendations, summary of reported intakes, and highlights future directions.

## Physiological Considerations For PARA-SCI

A SCI will affect body function and composition, metabolism, and energy expenditure (8). Furthermore, PARA-SCI include a diverse group with different etiologies and physiological characteristics (9). For example, an individual with an acute SCI will present differently than an individual with a chronic SCI or an individual with tetraplegia compared with paraplegia.

An examination of the physiological adaptations following the injury describes the impact of a SCI on the body. Post-injury, there are dramatic changes with a partial or complete loss of neurological (motor and sensory) function and changes in the activation of the autonomic nervous system below the level of the injury. Muscle atrophy is severe, with an estimated 40% reduction in total skeletal muscle cross-section area at 6 weeks and a further 20% 2 years post-injury, resulting in a potential 60% reduction in total lean muscle mass (10, 11). Mechanistically, the reduced muscle mass and increased fat mass, often referred to as secondary sarcopenia (12), has been linked to a breakdown in the excitation–contraction coupling in the skeletal muscle, reductions in protein synthesis, decreases in anabolic hormones (e.g., testosterone, growth hormone, and insulin-like growth factor-1), and increased proteolysis in addition to the immobilization (12, 13). Body composition assessments in individuals with SCI show increased total fat mass and reduced total fat free mass (14) as compared with able-bodied controls. PARA-SCI have higher body fat percentages and lower lean body mass in the legs and entire body compared with able-bodied controls (15, 16). Alterations in body composition in PARA-SCI result in a reduction in resting metabolic rate (5). Finally, “sit-forms” of exercise result in reduced energy expenditure during physical activity (8).

Skeletal muscle atrophy and increased sedentary time increase the risk of glucose intolerance, potentially leading to insulin resistance (17). Osteoporosis, oxidative stress, chronic systemic inflammation, reduced cardiovascular efficiency, dyslipidemia, and cardiovascular disease (CVD) risk are also elevated with a SCI (17). While physical activity can positively impact the aforementioned health concerns, CVD risk remains elevated in Paralympic athletes with a SCI (18). A more recent assessment comparing Paralympic athletes with an amputation vs. a SCI found that PARA-SCI with a high lesion SCI had a higher platelet-derived cardiovascular risk as compared with those with an amputation, which the authors attributed to malnutrition (17).

A SCI significantly affects the gastrointestinal (GI) tract, with GI complications accounting for ~11% of hospitalizations in individuals with a SCI (19). A SCI results in impaired colonic motility, vascular tone, and mucosal secretions (20). Individuals with a SCI often develop neurogenic bowel and an estimated 20–60% of the population experience changes in bowel function (19). Neurogenic bowel manifests as changes in the bowel physiology (e.g., colon motility and/or loss of anorectal sphincter function) and altered GI transit time (21). Often, this leads to constipation and impaction (19). In an effort to mitigate the constipating effects of neurogenic bowel, individuals with SCI may use suppositories, laxatives, and/or fiber supplements (20). A SCI can also increase the GI transit time (21), which may affect nutrient absorption and negatively impact mucosal function. In addition, changes in gut motility can lead to variations in the composition of the gut microbiota, creating a state of dysbiosis, whereby the balance between beneficial and pathogenic bacteria is negatively skewed (22). Furthermore, antibiotic use due to increased urinary tract infections, pneumonia, and pressure ulcers is elevated in this population (23). Consistent antibiotic use affects the beneficial gut bacteria in addition to the harmful strains, resulting in an unfavorable composition of the gut microbiota. Malnutrition, physical inactivity, and psychological stress can also exacerbate the effects of a SCI on the gut microbiome (24). Indeed, male patients with a SCI had increased gut dysbiosis as compared with an able-bodied control group (20). This creates a vicious circle, with a SCI causing gut dysfunction, leading to gut dysbiosis, subsequently impairing immune function, which, in turn, increases susceptibility to infections (22).

A SCI increases the risk of neurogenic bladder (25), whereby major urologic complications evolve including urinary tract infections, bladder diverticula, bladder stones, urethral trauma, bladder cancer, hydronephrosis, and renal failure. Athletes are at an elevated risk as for urinary tract infections and their presence impairs performance due to a loss of training days or withdrawal from competition (26). Another concern is upper GI dyspepsia, which is often treated with proton-pump inhibitors to reduce acid production (19). There is also evidence that sensitivity of vagal afferents to neuroactive peptides, neurotransmitters, and macronutrients may be diminished in a SCI (19), further complicating the matter. Finally, dysphagia, disordered swallowing function, is prevalent in this population, and consequently may affect food choices and eating habits (27). In conclusion, the physiological adaptations to the injury and its secondary complications may influence the protein needs of PARA-SCI.

## Role of Dietary Protein in Body Composition and Sport Performance as it Relates to PARA-SCI

Research regarding the role of protein in PARA-SCI is limited; however, the effects of protein on sport and exercise performance in able-bodied athletes are well-researched. The role of dietary protein in sport is multifactorial, affecting muscle protein synthesis (MPS), lean body mass, strength and power, energy production, and muscle damage/repair. With respect to body composition, skeletal protein turnover is a dynamic process that is highly responsive to exercise and dietary protein intakes. Muscle protein balance is negative in response to resistance exercise in the absence of feeding; however, it increases if amino acids are provided (28, 29). Protein/amino acid (AA) supplementation, in combination with resistance exercise, demonstrates a dose-dependent effect on MPS, with some suggesting 20 g of high-quality protein as optimal for able-bodied athletes (29). Mechanistically, compared with recovery from an acute bout of resistance exercise, in fasted or carbohydrate-fed state, protein supplementation results in higher activation of the mammalian target of rapamycin complex 1 (mTORC1), a crucial myocyte protein signaling MPS (30). In addition to promoting MPS, dietary protein may play a role in maintaining lean body mass, evidenced by studies looking at athletes aiming to lose weight while conserving muscle mass (31). Research regarding MPS and dietary protein in PARA-SCI is lacking; however, it is hypothesized that the aforementioned effects and mechanisms would apply to PARA-SCI in some capacity.

Performance wise, increased muscle strength was found when protein supplements were added to a resistance exercise program (32). The same systematic review concluded that protein supplementation could also improve aerobic and anaerobic power. The authors note, however, that the results were inconsistent in both cases (32). Protein also has a role in energy production. For example, leucine is an AA that can be oxidized during endurance exercise to a considerable extent (6). Studies have begun to explore the impact of protein on endurance exercise; however, results are inconclusive (28). Branched chain AA (BCAA)—leucine, isoleucine, and valine—intakes during exercise reduce perceptions of central fatigue and perceived exertion, and increase mental performance; however, other studies fail to support these improvements (33). Finally, protein combined with carbohydrate intake during ultra-endurance exercise may reduce subjective measures of muscle soreness and markers of muscle damage (28). Protein supplementation is also likely to be beneficial for PARA-SCI with respect to anaerobic power and possibly aerobic performance; however, insufficient evidence is available to make any strong conclusions.

To summarize, dietary protein plays an important role in muscle hypertrophy and remodeling, maintenance of lean body mass, and the optimal functioning of metabolic pathways. Emerging evidence suggests that protein has additional roles in exercise performance and training adaptations, which require further investigation.

## Protein Recommendations for Able-Bodied Athletes With Relevance to PARA-SCI

Determining protein needs for PARA-SCI is complex; consequently, protein recommendations for this population are not well-established nor is there universal agreement. Considering these limitations, we will outline the guidelines established for able-bodied athletes with relevance to PARA-SCI.

Protein recommendations for athletes should take into account the frequency, intensity, and type of exercise, with athletes focused primarily on strength sports and those desiring muscle hypertrophy typically requiring the highest amounts. The athlete's goals, as well as, their training phase are also critical considerations. Increasingly, there is a focus on periodized nutrition, whereby intakes are matched to the specific training sessions within a periodized plan rather than a general classification as strength or endurance athlete (34). All considered, however, recommendations for all athlete types are elevated as compared with non-athletes (34). Many of these same principles will likely apply to PARA-SCI, and there is evidence to suggest that individuals with a SCI experience muscle hypertrophy in response to exercise (35). However, there is little evidence in PARA-SCI athletes, and the role of dietary protein in MPS has not been determined in this population. While a similar response to able-bodied athletes is hypothesized, confirmation is required, and there is the possibility of differing responses including magnitude and amount of time required to see an effect.

Protein recommendations are made with respect to the quantity and timing of protein intakes. The recommended daily allowance (RDA) of the able-bodied population is 0.8 g/kg body mass per day (36). In able-bodied athletes, the amount of protein required each day ranges from 1.2 to 2.0 g/kg body mass (34). Typically, it is advised to space protein meals about 3 h apart, as well as before and after strenuous exercise (28). Importantly, even higher protein intakes, ranging from 2.3 to 3.1 g/kg body mass, may be advised for short time periods in resistance-trained athletes, when overall energy intakes are being reduced (e.g., during weight loss) (31). Endurance athletes are recommended to consume between 1.6 and 2.4 g/kg protein during caloric restriction (31). Conversely, protein intakes above 2.0 g/kg body mass in weight-stable, endurance athletes do not appear to improve performance (28). With respect to individual servings, for MPS, generally 0.25 g/kg of body weight or 20–40 g of protein is suggested (28). Further research is required to determine if these recommendations are relevant to PARA-SCI, while, as of yet unstudied, it is interesting to consider if protein recommendations based on total body mass are appropriate for PARA-SCI, as they are for able-bodied athletes, or if lean muscle mass would be more appropriate. Furthermore, the importance of acquired vs. congenital lesions and variance in functional muscle mass may affect protein recommendations.

The timing of protein intake is a key factor in optimizing performance, as MPS is upregulated for at least 24 h post-resistance training, and there is an increased sensitivity to dietary protein intakes during this time (34). Practically, protein intakes tend not to be evenly spaced throughout the day, and are often insufficient at breakfast and excessive in later meals. To take advantage of the increased sensitivity, multiple protein-containing meals and snacks post-exercise and during the day are advised. These recommendations hold true for all types of exercise, even if muscle hypertrophy is not the athlete's primary goal (34). Furthermore, the intake of protein or amino acid combinations before and during resistance exercise will maximize muscle repair and hypertrophy (6, 7, 28). While these recommendations seem reasonable for PARA-SCI, to our knowledge, spacing and protein timing, as it relates to MPS, has not been studied in this population. Less is known regarding the potential benefit, or harm, of protein intake before and during endurance exercise. It is suggested that protein feeding during exercise might help maintain a favorable anabolic hormone profile, minimize increases in muscle damage, and increase time to exhaustion during prolonged running and cycling (28). Notably, time trials are considered to be a better indicator of performance than time to exhaustion tests. Here, a meta-analysis reported favoring a carbohydrate–protein combination; however, the individual studies were mixed (37). Given the potential for reduced muscle soreness, a recent position statement by the International Society of Sports Nutrition recommends 0.25 g/kg body mass of protein per hour of endurance exercise in combination with carbohydrate (28); however, caution is advised due to the potential for gastrointestinal discomfort. The applicability of dietary intakes during exercise for athletes with a SCI is questionable given the increased challenges regarding the gastrointestinal system. Finally, the potential benefits of protein intake pre-sleep have been investigated, and evidence suggests 30 g of protein, particularly casein, before going to bed may stimulate MPS and recovery (28). The effects of pre-sleep protein on PARA-SCI would be of interest, as this is an intervention that could be incorporated into daily routines.

Standard protein recommendations for athletes following a purely plant-based diet may not be optimal. Lynch et al. (38) and Rogerson (39) recommend increasing protein intake in vegan athletes up to 1.7–2.0 g/kg body mass, with an even higher amount during weight loss or energy restriction (e.g., up to 2.7 g/kg). A higher intake might be required to include a sufficient amount of essential AA through their diet. Furthermore, a wider variety of food sources seems to be needed to obtain this goal. The American Dietetic Association concluded, however, that if caloric requirements are met, protein or AA needs should be attained for vegan or vegetarian athletes (40). Studies assessing the effects of vegan or vegetarian PARA-SCI athletes are unavailable to our knowledge.

## Protein Recommendations for PARA-SCI

**Figure 1:**
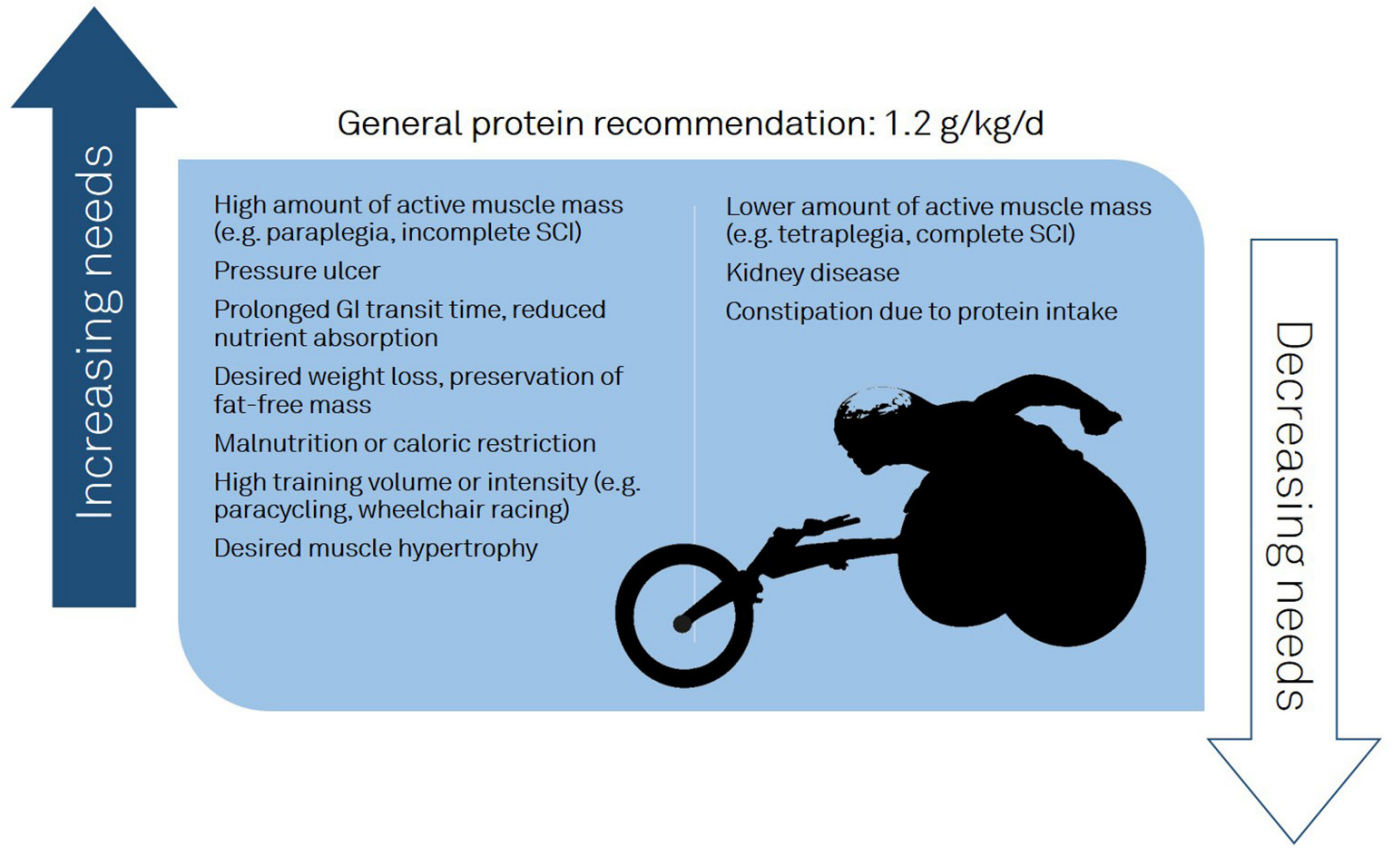
Factors influencing protein recommendations in athletes with a spinal cord injury.

Protein intakes for PARA-SCI are undetermined with Goosey-Tolfrey et al. noting “there is a dearth of reported literature assessing the protein or amino acid requirements for athletes with SCI” (41). While there are recommendations for able-bodied athletes and limited guidelines for individuals with a SCI, PARA-SCI have received less attention. As PARA-SCI maintain active muscle mass in the upper body, we can imagine a similar response to strength training and MPS as shown in able-bodied athletes. We must acknowledge, however, that intervention trials, with a sufficient sample size, investigating the effect of strength training and protein supplementation on the stimulation of MPS in PARA-SCI are lacking. Considering the aforementioned physiological factors associated with a SCI, and the effect of sport on protein needs; we postulate that neither the able-bodied athlete recommendations nor non-athlete SCI recommendations be directly transposed onto the PARA-SCI population. In addition, athletes with SCI compete in different sports, which have different energy demands. Training volume, intensity, and exercise type as well as secondary conditions (e.g., gastrointestinal motility, pressure ulcer) will affect protein demands (Figure 1). Finally, the heterogeneity of PARA-SCI with respect to the level of injury, injury type (e.g., complete or incomplete), and the time since injury (5) adds additional challenges.

If one extrapolates from able-bodied athletes, the logical assumption would be to propose increased protein needs for optimal performance; however, rigorous scientific study is required to determine if the able-bodied recommendations are safe/appropriate or how they need to be adjusted.

Importantly, there are several factors unique to PARA-SCI that indicate caution before universally recommending higher intakes. The first consideration is the impact of total energy expenditure (TEE) on protein intakes. While a few studies have looked at estimating TEE in PARA-SCI (8, 42), additional work is required, especially in females. Ambiguity surrounding TEE is a barrier to the determination of protein needs, as it makes it difficult to determine the appropriate overall energy intake and subsequently protein needs. Regardless, the available evidence would suggest lower TEE in PARA-SCI than able-bodied athletes (5, 8), necessitating reduced food intakes. Reductions in TEE will make it difficult to obtain sufficient protein in the context of the overall macro- and micronutrient balance. For example, if energy intakes in PARA-SCI are lower than able-bodied athletes, due to reduced TEE, and protein recommendations remain unchanged, fat and/or carbohydrate intakes will need to be reduced. The importance of this is highlighted in context of elevated body fat found in PARA-SCI (11). Second, neurogenic bladder and associated complications could have implications on dietary protein intakes (e.g., decreased intake), as it seems possible that a high-protein diet might have some negative consequences in patients with kidney disease (43). In addition, bladder overactivity treated with anticholinergics can further diminish GI motility and affect diet due to food–drug interactions (44). Athletes should undergo a yearly medical screening including a check of bladder and kidney function, if they are considering a higher-protein diet. Finally, the impact of neurogenic bowel disease and gut dysbiosis should be considered, as this could affect fiber and hydration needs, ultimately affecting the overall diet and food choices.

The impact of high-protein diets, on the metabolic health of those with a SCI, is of interest; however, little is known. A recent study found that in five individuals with a long-standing SCI, an isocaloric high-protein diet (30% of total energy) resulted in reduced fat mass with no change in lean mass after an 8-week intervention (45). An additional study looking at a low-carbohydrate/high-protein diet is underway (46). To our knowledge, high-protein diets have not been tested in PARA-SCI. It should be noted, however, that studies suggest athletes with a SCI are at risk for overall low energy availability (47) and increased protein intakes could help when athletes are in caloric restriction or during phases of intended weight loss.

Another key factor in considering protein recommendations for PARA-SCI is pressure ulcers. Individuals with a SCI are at a high risk of developing pressure ulcers, which increases protein needs for wound healing (48). Conversely, nutritional factors, such as malnutrition and anemia, can further increase the risk of pressure ulcers (49). The treatment guidelines concerning nutritional therapy include a protein intake of 1.25–2.0 g/kg body mass for adults with a chronic SCI and a pressure ulcer or at risk to be malnourished, and 2.0 g/kg body mass for individuals with an acute SCI (50). Furthermore, the guidelines recommend the use of high-caloric, high-protein fortified foods or nutritional supplements in patients with a high risk for malnutrition or an existing pressure ulcer, especially if nutritional needs cannot be achieved through normal dietary intakes. In addition, the review states that arginine supplementation might be beneficial for the treatment of pressure ulcers (50). Brewer et al. (51) found supplementation with 9 g of arginine during the healing phase of a pressure ulcer significantly reduced the time required to heal as compared with controls from retrospective data (10.5 ± 1.3 weeks vs. 21.0 ± 3.7 weeks). In addition, a recent systematic review (52) revealed that arginine could potentially improve wound healing in malnourished and non-malnourished able-bodied individuals with a pressure ulcer. Considering the evidence available, arginine supplementation could be possibly applied in addition to the other nutritional guidelines (50).

At present, we are unable to present robust scientific evidence for protein intakes in PARA-SCI; however, we can extrapolate from recommendations for those with a SCI and able-bodied athletes. Evidence would indicate that in individuals with a chronic SCI, 0.8–1.0 g/kg/day of protein should be sufficient to achieve protein balance (53). Given that there is significant evidence that able-bodied athletes require elevated protein intakes, it is reasonable to assume an increased need in PARA-SCI as well. Furthermore, a minimum of 1.25 g kg BW is recommended for those with a SCI with pressure ulcers, suggesting this amount is safe for the SCI population (50). All considered, it would be reasonable to recommend 1.2 g kg BW as a minimum (Figure 1), particularly for those desiring muscle hypertrophy and those with a high TEE (e.g., high training volume/intensity, large amount of active muscle mass).

## Protein Intake Reported

Nutrient intake in individuals with a chronic SCI has been reported in previous studies (5). Table 1 provides a summary of protein intakes in individuals with a SCI. Data are presented as total daily protein intake and protein intake per kilogram body mass. Total protein intakes ranged from 56 to 96 g/day in females and 63 to 95 g/day in males (Table 1). Generally, protein intakes were above the recommended intake of 0.8 g/kg body mass. Doubelt et al. (56) mention that 25% of the participants had intakes below 0.66 g/kg body mass, which corresponds to the estimated average requirement described by them. In addition, Gorgey et al. (57) showed an intake below 0.8 g/kg body mass in manual wheelchair users. When nutrient intakes in individuals with an acute or chronic SCI were compared, Perret and Stoffel (65) found no significant differences in protein intakes. Differences in protein intake between individuals with paraplegia or tetraplegia per kilogram body mass were not consistently observed; nor was gender a factor. However, the limited available literature does not allow any differentiated distinction between protein intake based on lesion level (e.g., para- vs. tetraplegia) nor between men and women. In conclusion, the majority of this population exceeded the RDA of 0.8 g/kg body mass for healthy adults and individuals with a SCI, suggesting protein intakes are adequate in the general SCI population.

**Table 1:** Protein intake in individuals with chronic SCI (non-athletes).

**References**	**Subjects**	**Number of subjects**	**Methods**	**Daily protein intake**
Allison et al. (54)	Anti-inflammatory diet in chronic SCI:	20 (*n* = 12 intervention group, eight control group)	7-day food diary at baseline, 3-day food diary at 1, 2, and 3 months	Intervention group: Baseline 73 ± 24 g/day^a^ 3 months: 95 ± 22 g/day^a^ No data for control group shown
Barboriak et al. (55)	Individuals with a SCI (15 with paraplegia, 22 with tetraplegia)	37	24-h recall and checking and weighing leftover meals	Paraplegia: 95 ± 32 g/day = 1.3 g/kg/day Tetraplegia: 86 ± 28 g/day = 1.2 g/kg/day
Doubelt et al. (56)	Individuals with SCI (22 with tetraplegia, 12 with paraplegia, 94% male)	34	Food frequency questionnaire	82 g/day = ~1.0 g/kg/day; 25% of the participants below 0.66 g/kg/day
Gorgey et al. (57)	Men with chronic motor complete SCI (10 with paraplegia, six with tetraplegia)	16	5-day food dietary log for 4 weeks	Manual wheelchair users: 65 g/day = ~0.75 g/kg/day
Gorgey et al. (58)	Chronic SCI, five participants resistance training (RT), four participants in the control group (C)	9	Daily food diary for 12 weeks	RT: 1.1 ± 0.29 g/kg/day C: 1.09 ± 0.24 g/kg/day
Groah et al. (59)	Individuals with SCI (24 males with tetraplegia, 37 males with paraplegia, one female with tetraplegia, 11 females with paraplegia)	73	4-day food log	Male tetraplegia: 85.7 g/day^a^ Male paraplegia: 87.6 g/day^a^ Female tetraplegia: 95.5 g/day^a^ Female paraplegia: 75.3 g/day^a^
Javidan et al. (60)	Patients with SCI (217 male, 48 female)	265	24-h dietary recall interviews	69.6 g/day = 1 g/kg/day 86.8% below 1.5 g/kg/day
Levine et al. (61)	Individuals with chronic SCI (24 male and 9 female)	33	7-day dietary record and a food frequency chart	Male: 69 g/day^a^ Female: 56 g/day^a^
Lieberman et al. (62)	Individuals with chronic SCI	100	Food frequency questionnaire	100.3 g/day^a^
Nightingale et al. (63)	Individuals with paraplegia	33	Weighted food diary for 7 days	74.6 g/day = 0.98 g/kg/day
Pellicane et al. (64)	Inpatient rehabilitation (eight with paraplegia, eight with tetraplegia)	16	After meals, calculation of energy intake by examining food trays	All SCI: 71.5 ± 25.0 g/day Paraplegia: 0.86 ± 0.37 g/kg/day Tetraplegia: 0.92 ± 0.43 g/kg/day
Perret and Stoffel-Kurt (65)	Patients with an acute and a chronic SCI	24 (12 per group)	Daily food diary for 7 days	Acute: 74.6 ± 10.0 g/day = 1.07 g/kg/day Chronic: 71.4 ± 7.9 g/day = 1.07 g/kg/day
Sabour et al. (66)	Individuals with a chronic SCI	162	Semiquantitative food frequency questionnaire	Complete SCI: 64. 7 ± 23.2 g/day^a^ Incomplete SCI: 64.3 ± 24.9 g/day^a^ Tetraplegia: 63.3 ± 23.7 g/day^a^ Paraplegia: 65.4 ± 25.4 g/day^a^
Tomey et al. (67)	Individuals with a chronic SCI	95	Semiquantitative food frequency questionnaire	82.3 ± 31.7 g/day^a^
Walters et al. (68)	Individuals with a chronic SCI (63 males, 14 females)	77	Multiple-pass 24-h recalls	Male: 81.8 g/day = ~1.03 g/kg/day Female: 70.9 g/day = ~1.0 g/kg/day

*SCI, spinal cord injury*.

a *Body mass not reported*.

Protein intakes in PARA-SCI are presented in Table 2. Relative protein intakes ranged from 1.1 to 1.9 g/kg body mass. Gender differences are inconclusive, as Gerrish et al. (71) and Madden et al. (75) showed higher intakes in male athletes; whereas Krempien and Barr (74) found higher intakes in female athletes. Furthermore, the time point of the season at which protein intake was assessed seemed to influence the intake (70, 71, 73). Possible explanations for the effect of season include differences in training intensity, training focus (e.g., strength vs. endurance), or total training hours (e.g., difference in energy expenditure).

**Table 2:** Protein intake in athletes with SCI.

**References**	**Subjects**	**Number of subjects**	**Methods**	**Daily protein intake**
Eskici and Ersoy (69)	Female wheelchair athletes	22	24-h retrospective diet recall	Women 1.6 ± 0.3 g/kg/day
Ferro et al. (70)	Male elite wheelchair basketball players	11	3-day food-weighing diary in 2 months during the pre-competitive period	May: 1.7 ± 0.6 g/kg/day June: 1.5 ± 0.5 g/kg/day
Gerrish et al. (71)	Canadian and US elite wheelchair athletes (tennis, track, basketball, and rugby)	19 women 20 men	Self-reported, single 24-h food journal in autumn and winter	**Autumn:** Women 1.1 ± 0.3 g/kg/day Men 1.7 ± 0.2 g/kg/day **Winter:** Women 1.3 ± 0.4 g/kg/day Men 1.3 ± 0.3 g/kg/day
Goosey-Tolfrey and Crosland (72)	Wheelchair Games player	14 women 9 men	7-day food-weighing diary over seven consecutive days	Women: 1.00 ± 0.29 g/kg/day Men: 1.37 ± 0.33 g/kg/day
Grams et al. (73)	Male wheelchair basketball players (5 amputees, 12 with SCI)	17	3-day weighed food journal over three consecutive days during three training camps over two consecutive years	**Training camp 1** 1.6 ± 0.7 g/kg/day **Training camp 2** 1.5 ± 0.5 g/kg/day **Training camp 3** 1.9 ± 0.7 g/kg/day
Krempien and Barr (74)	Elite athletes with a spinal cord injury	8 women 24 men	3-day self-reported food journal kept at home and training camp	**Training camp:** Women 1.7 ± 0.3 g/kg/day Men 1.4 ± 0.4 g/kg/day **Home:** Women 1.6 ± 0.6 g/kg/day Men 1.3 ± 0.4 g/kg/day
Madden et al. (75)	Various different wheelchair sports, mainly wheelchair basketball	22 women 18 men	3-day, consecutive self-reported food journal	Women 1.4 (1.1–1.6) g/kg/day Men 1.6 (1.4–2.2) g/kg/day

*SCI, spinal cord injury*.

In comparison with the data presented in Table 1, protein intakes based on body mass appear to be higher in PARA-SCI compared with the general population with a SCI. A comparison with able-bodied athletes is difficult given variability in sport and the large range in reported intakes. However, a benchmark could be found in a Dutch study, including 553 data sets from different sports, where 80% of able-bodied athletes met the minimum recommendation of 1.2 g/kg body mass (76). To summarize, PARA-SCI typically meet or exceed the minimum able-bodied athlete recommendations for protein and intakes fall within a similar range.

## Protein Supplementation in SCI

Research in able-bodied athletes indicates protein supplementation (e.g., whey) following a strength session or high-intensity interval training could increase post-exercise MPS, thereby enhancing training stimulus, recovery, and training adaptation (7, 28, 31). Only a few studies have investigated the effects of protein supplementation in individuals with a SCI (77–79). Kressler et al. (78) divided 11 individuals with a cervical lesion level (male and female) into two groups and supplemented them with whey protein immediately after a circuit training or on a rest day. Performance in a one-maximum repetition test significantly increased in both conditions, but anaerobic capacity and fatigue resistance might have been further enhanced in the circuit-training group. A very similar study (77) showed no effect of protein supplementation on fat oxidation. Both studies seemed to be underpowered, as evidenced by the small sample size; therefore, it is difficult to draw any definitive conclusions. Nash et al. (79) supplemented three subjects with either a whey + carbohydrate or a soy supplementation + sweetener in individuals with a lesion between C5 and T4 during ambulation. Some positive effects on time or distance of ambulation can be shown with the whey + carbohydrate compared with soy supplementation, but again, the results are limited by the small sample size. A similar conclusion was made by Navarrete-Opazo et al. (80) in their review article on protein supplementation in individuals with a SCI, as those studies show a risk of bias, small sample sizes, and missing data. Consequently, it would be premature to comment on the effectiveness of protein supplementation in PARA-SCI.

## Future Directions

The field of performance nutrition in PARA-SCI is emerging and there is a need for future studies regarding optimization of dietary protein intakes. Going forward, nitrogen balance studies may be beneficial in determining overall protein needs in PARA-SCI, as they have been used in able-bodied athletes (81). Protein timing, dosage, and quality require exploration in the context of MPS and muscle adaptation in PARA-SCI. The safety of elevated doses as well as appropriate screenings and protocols also need to be established in this population. Due to the effects of a SCI on the GI system (e.g., transit time) and nutrient absorption, the dosage and timing might play a role. Furthermore, studies measuring muscle hypertrophy and strength in response to protein intakes in PARA-SCI are also required, as nitrogen balance alone will likely be insufficient to determine optimal levels for performance (6). Critically, although challenging, the aforementioned studies will need to be undertaken with sufficiently large samples sizes and scientific rigor to ensure confidence in the conclusions. Finally, the available research does not adequately consider the impact of acquired vs. congenital lesions, lesion level or completeness, and variance in functional muscle mass. A breakdown of the role of protein and recommendations by these factors would be valuable.

## Conclusion

To conclude, determining protein intakes for PARA-SCI will be challenging as optimal physical performance and physiological changes associated with a SCI need to be considered. The heterogeneity of the population will further complicate recommendations. The lower energy demands of a PARA-SCI and risk of experiencing secondary complications such as pressure ulcers (increased protein demands) or a kidney disease (reduced protein intake) may also alter protein recommendations. Future studies are needed to determine the effectiveness and safety of protein supplementation in PARA-SCI. While barriers exist to determining recommendations, they are essential and these challenges do not excuse the scientific community from working with PARA-SCI. Presently, in the absence of clinical trials, it seems prudent to recommend that PARA-SCI consume a minimum of 1.2 g/kg body mass, as this has been established as a minimum recommendation for able-bodied athletes. It can be assumed that intakes at this level will be well-tolerated in this population, as amounts in this range are commonly recommended for individuals with a SCI experiencing pressure ulcers.

## Author Contributions

JP and JF: conceptualization, writing—original draft, and writing—review and editing. Both authors contributed to the article and approved the submitted version.

## Conflict of Interest

The authors declare that the research was conducted in the absence of any commercial or financial relationships that could be construed as a potential conflict of interest.
